# Optimization Method for Wide Beam Sonar Transmit Beamforming

**DOI:** 10.3390/s22197526

**Published:** 2022-10-04

**Authors:** Louise Rixon Fuchs, Atsuto Maki, Andreas Gällström

**Affiliations:** 1 Division of Robotics, Perception and Learning, KTH Royal Institute of Technology, SE-100 44 Stockholm, Sweden; 2 Saab Dynamics, SE-581 88 Linköping, Sweden

**Keywords:** autonomous underwater vehicles, sonar, phased antenna arrays, transmit beamforming, convex optimization, beampattern, side-scan sonar, forward-looking sonar, seabed mapping

## Abstract

Imaging and mapping sonars such as forward-looking sonars (FLS) and side-scan sonars (SSS) are sensors frequently used onboard autonomous underwater vehicles. To acquire information from around the vehicle, it is desirable for these sonar systems to insonify a large area; thus, the sonar transmit beampattern should have a wide field of view. In this work, we study the problem of the optimization of wide transmission beampatterns. We consider the conventional phased-array beampattern design problem where all array elements transmit an identical waveform. The complex weight vector is adjusted to create the desired beampattern shape. In our experiments, we consider wide transmission beampatterns (≥20${}^{\circ }$) with uniform output power. In this paper, we introduce a new iterative-convex optimization method for narrowband linear phased arrays and compare it to existing approaches for convex and concave–convex optimization. In the iterative-convex method, the phase of the weight parameters is allowed to be complex as in disciplined convex–concave programming (DCCP). Comparing the iterative-convex optimization method and DCCP to the standard convex optimization, we see that the former methods archive optimized beampatterns closer to the desired beampatterns. Furthermore, for the same number of iterations, the proposed iterative-convex method achieves optimized beampatterns, which are closer to the desired beampattern than the beampatterns achieved by optimization with DCCP.

## 1. Introduction

Sonar systems are remote sensing systems used for determining the presence of objects or geometrical structures in the sea. Autonomous underwater vehicles (AUVs) are often equipped with sonar systems for tasks such as obstacle avoidance and seabed mapping. Active sonar involves the transmission of an acoustic sound beam, where the reflections on underwater targets are then received and processed [1]. The sonar produces the transmission beam through the spatial filtering technique beamforming [2]. Beamforming is essential in several fields such as radar, sonar, communications, imaging, biomedical, geophysical exploration, and astrophysical exploration [3]. A recent review [4] covers the progress of array synthesis for phased antenna arrays in the wireless communication and remote sensing domain.

Beamforming aims to design a beampattern suitable for the desired application. For conventional sonar systems, it is desirable to send out a wide transmission beampattern (>20${}^{\circ }$). Aide transmission beampattern is of importance for sonar in several different applications. Some of these applications are Forward-Looking sonars (FLS) for obstacle avoidance [5], seabed mapping with side-scan sonars (SSS) [6], Synthetic Aperture sonar Systems (SAS) [7], and acoustic navigation aid using Compact Correlation Velocity Logs (CVLs) [8]. In its simplest form (delay-and-sum), the beamformer linearly combines spatially sampled time-series signals into a scalar output (a beampattern). For narrowband signals, the beamformer is described by [3]:

(1) $$y\left(k\right)=\sum_{i=1}^{N}w_{i}^{H}x\left(k\right),$$

where w is the weight vector, *k* is the time, x(k) is the data vector, y(k) is the beampattern, *H* is the Hermitian, and *N* are the sensor elements. The beampattern can be designed for an array of elements by choosing the complex weights w, which determine both the amplitude and phase of the beampattern [9]. The larger part of the literature on sonar beamforming discusses receive beamforming, where it is usually desirable to create narrow beams in the direction of a target and achieve as low side-lobe levels as possible. For passive sonar, direction of arrival (DOA) estimation is a problem, where adaptive methods such as Minimum Variance Distortionless Response (MVDR) are frequently used [9,10,11].

Other advanced methods for beamforming also exist, such as Linear Constrained Minimum Variance Beamforming (LCMV) [12], MUltiple SIgnal Classification (MUSIC) [13], Compressive sensing (CS) [14,15], Fast Fourier Transform (FFT) [16], and convex optimization [17]. In this work, we focus on convex optimization. The convex optimization-based methods have several advantages [18], such as being deterministic, solvable in polynomial time, and easy to implement with available off-the-shelf solvers, such as CVXOPT [19]. A local optimum found with convex optimization is also a global optimum, ensuring the best solution. Convex optimization methods have mostly been used for designing beampatterns in radar and communication [9,17,20]. In [9], convex optimization was used for acoustics arrays. To the best of our knowledge, there are relatively few papers dealing with optimization for sonar transmit beamforming [21]. However, beamforming optimization is a general field with applications in several areas [17,22,23,24,25,26,27,28]. While convex optimization has been successfully used in other areas, its use for wide beam transmit beamforming remains to be explored.

The purpose of this work is to introduce an iterative-convex optimization technique suitable for imaging sonars that need to transmit wide beams that cover large areas of seafloor. The optimized beamformer is meant to be optimized offline on simulated data and then used for a sonar imaging system onboard an AUV. Our contribution in this paper is an iterative-convex optimization technique for wide transmission beam design. We consider the conventional phased-array beampattern design problem, where all array elements transmit an identical waveform. The complex weight vector is then adjusted to create the desired beampattern shape. In our examples, we consider narrowband signals. The novelty in our method is that it needs fewer iterations than the baseline methods to converge to the desired beampattern. The proposed method draws inspiration from previous work on antenna array pattern synthesis [16]. In contrast to [16], we use convex optimization instead of a fast Fourier transform (FFT). The novelty in this work also lies in studying the convex optimization problem for wide sonar transmit beams, which have not been studied in the literature.

The outline of this paper is as follows: In Section 2, we cover the basic terminology related to beamforming with a special focus on optimization-based methods, and we introduce our proposed method. In Section 3, we present our experimental results. Finally, our discussion and conclusions are given in Section 4.

## 2. Materials and Methods

### 2.1. Transmission Pattern Optimization

Transmission pattern optimization deals with designing the optimal transmission beampattern given a specification for the desired beampattern mask. The far-field beampattern for a linear array of *N* elements, which transmits a narrow-band plane wave, can be defined as:

(2) $$G\left(\theta \right)=\sum_{n=1}^{N}w_{i}exp\left(-j\frac{2\pi }{\lambda }x_{i}\cos\theta \right),$$

where θ is the transmission direction for the wave, λ is the wavelength, and $x_{i}$ is the location of the antenna array element *i*. The signal outputs from the *N* elements are weighted by the complex weights $w_{i}$. The optimization task is to find the weights $w_{i}$, with amplitudes and phase that give the desired beampattern, which we write as $BF_{tar}$. The complex weights $w_{i}$ determine the shape of the desired beampattern. For many sonar transmit beampatterns, it is desirable to insonify a large area.

### 2.2. Beampattern Mask Design

**Figure 1:**
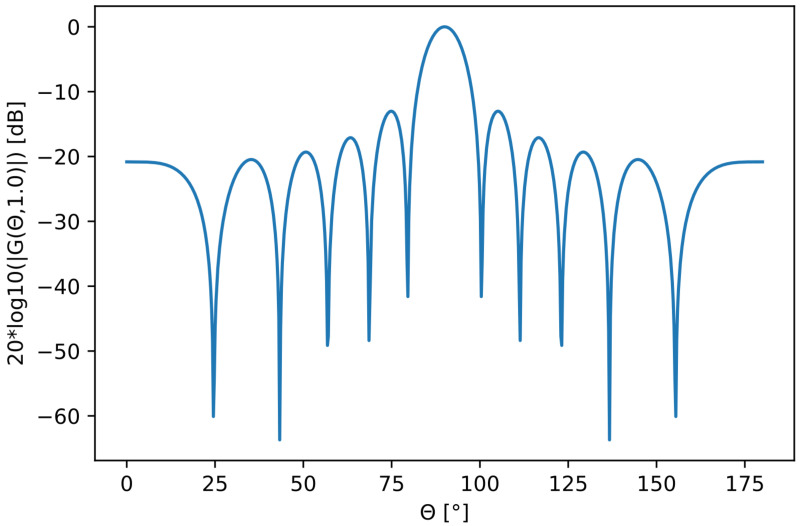
Beampattern (blue line) for the 11-element linear phased array.

The energy in the main lobe is distributed in a narrow-angle interval in the center (see Figure 1). The half-power point where the antenna gain has fallen −3 dB from its peak value defines the main lobe beamwidth. We designed the desired beampattern mask for our ideal transmission beampattern. This beampattern mask consisted of two parts:

(3) $$BF=BF_{tar}+BF_{sup},$$

where $BF_{tar}$ was the desired main lobe, and $BF_{sup}$ was the desired sidelobe suppression. The amplitude values of the mask describe the amplification of the beam in a given direction. Here, we give an example of the beampattern mask design:

(4) $$\left\{\begin{array}{c} BF_{sup}:10^{-20/20},\forall \theta \\ BF_{tar}:10^{-1/20},\theta \in \left[\theta_{0},\theta_{N}\right] \end{array}\right.\;$$

We obtained the full mask by combining the two contributions:

(5) $$BF=BF_{tar}+BF_{sup}$$

**Figure 2:**
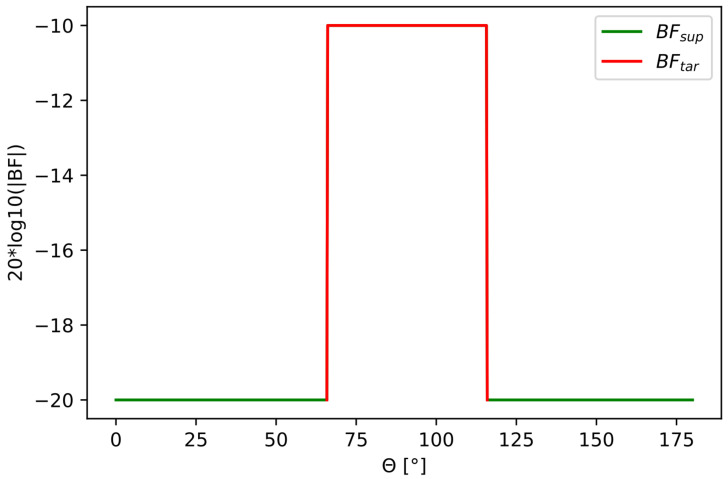
Beampattern mask including $BF_{tar}$ in red and $BF_{sup}$ in green.

The beampattern mask BF with its two contributions $BF_{tar}$ and $BF_{sup}$ are shown in Figure 2.

When choosing the amplitude value for the $BF_{tar}$ contribution, we must consider the available output power for the beampattern G(θ) in that interval. Looking at Figure 1, if we want to design a broader main lobe for $BF_{tar}$, we need to distribute the energy in a broader angle interval. Consequently, this will result in a lower maximum peak height. To tune the amplitude values of $BF_{tar}$ to an amplitude height that preserves the available energy of the beampattern G(θ), we performed the following energy tuning procedure:

1. We took the angle interval of $BF_{tar}$ that is $\left[\theta_{i},\theta_{N}\right]$;
2. We took the sum of the amplitude values of the beampattern in Figure 1: $\sum_{\theta =\theta_{i}}^{\theta_{N}}20\log_{10}\left|G\left(\theta \right)\right|$;
3. We manually fine tuned by balancing the amplitude values of $BF_{tar}$ so that the “energy’’ approximately matched:
  (6) $$\sum_{\theta =\theta_{i}}^{\theta_{N}}20\log_{10}\left|G\left(\theta \right)\right|\approx \sum_{\theta =\theta_{i}}^{\theta_{N}}BF_{tar}\left(\theta \right).$$

### 2.3. Convex Optimization

Convex optimization problems are optimization problems where the objective function is a convex function. A function $f:C^{n}\to R$ is convex if its domain is a convex set and for all $x,y\in C^{n}$ and all λ∈[0,1], we have:

(7) $$f_{i}\left(\lambda x+\left(1-\lambda \right)y\right)\leq \lambda f_{i}\left(x\right)+\left(1-\lambda \right)f_{i}\left(y\right).$$

A convex optimization problem can be written as [29]:

(8) $$\begin{array}{cc} \min\; & f_{0}\left(x\right)\\ s.t.\; & f_{i}\left(x\right)\leq b_{i},\;i=1,\ldots ,m \end{array}$$

where

- $x\in R^{n}$ is the optimization variable.
- $f_{0}\left(x\right)$ is the convex objective function.
- $f_{i}$ are convex functions.

If we write the beampattern as $G\left(\theta \right)=A\left(\theta_{tar}\right)w$, we can write our beampattern optimization problem as a convex optimization problem in the following way:

(9) $$\begin{array}{cc} \; & \min_{w}||A\left(\theta_{tar}\right)w-BF_{tar}\left|\right|\\ \; & |w_{i}|\leq 1.0\\ \; & |A\left(\theta_{sup}\right)w|\leq BF_{sup}. \end{array}$$

**Figure 3:**
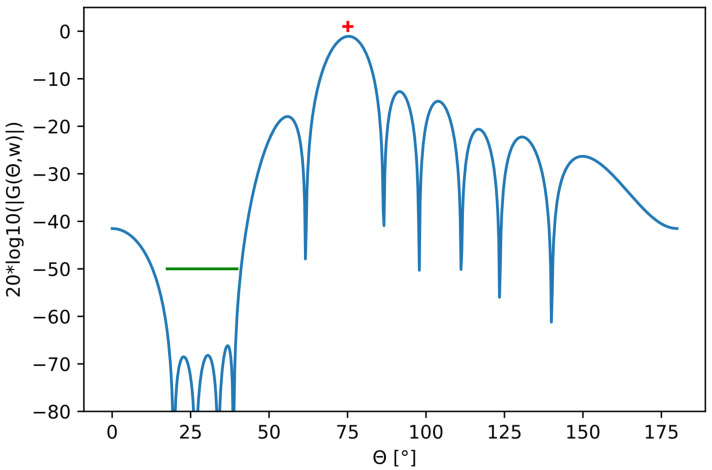
Example of optimized beampattern (blue) with suppression region marked out in green. The red cross is the direction of the beampattern maximum.

The optimization variable is the weight vector *w*, $\theta_{tar}$ is the desired transmit direction, $A(\theta_{tar})w$ is the optimized beampattern, and $\theta_{sup}$ is the angular interval where we want the beampattern to be suppressed. Figure 3 shows an example of an optimized beampattern with the attenuation $BF_{sup}$ marked out as a straight green line. To ensure that the optimization is convex, $BF_{tar}$ is a constant. The absolute value of the complex weight $w_{i}$ is set as less than or equal to one to fulfill the unit modulus constraint.

### 2.4. Disciplined Convex–Concave Programming (DCCP)

If we relax the constraint on BF to be constant, and we instead allow its phase to change while keeping the amplitude constant, we cannot solve the problem by convex optimization. However, one feasible approach is to use concave–convex optimization.

We used disciplined convex–concave programming (DCCP) [30] to solve the concave–convex optimization problem. DCCP makes use of a combination of disciplined convex programming (DCP) with convex–concave programming. For our implementation, we used the DCCP package, which is built on top of CVXPY [31]. The optimization problem is written the same way as the convex optimization case; the difference is that the absolute value of $BF_{tar}$ is constant, while the phase values may change during the optimization.

### 2.5. Iterative-Convex Method

We introduced an iterative-convex optimization method. The main steps in our algorithm were the following:

1. Choose the design of the desired beampattern $BF_{tar}$ and $BF_{sup}$.
2. Choose the learning rate α.
3. Choose the number of iterations *i* to run. Iterate steps 4–5 for *i* times.
4. Run convex optimization and obtain the new weight vector w. The problem is defined as in Equation (9).
5. Update $BF_{tar}^{i+1}$ as:
  (10) $$BF_{tar}^{i+1}=BF_{tar}e^{jArg\left(A_{tar}^{T}w^{i-1}\right)+\alpha \left(Arg\left(A_{tar}^{T}w^{i}\right)-Arg\left(A_{tar}^{T}w^{i-1}\right)\right)}$$

Note that the update in step 5 was conducted by comparing the two different phases for $w^{i}$ and $w^{i+1}$. Our method is convex when solving for the weight vector, but just as in concave–convex optimization, we allowed the phase to be complex in step 5. We ran the experiments with the learning rate set to α≤1.

Our method was inspired by the iterative method in [16]. However, in contrast to their work, we used convex optimization instead of an FFT.

### 2.6. Impact of Quantization Errors

We performed an analysis of the limited accuracy in bits of the amplitude and phase value. This was implemented similar to that in [32]. The impact of the frequency properties of the transmitting elements was not included in our analysis. Taking these properties into account may lead to an increased requirement on the quantification bit resolution.

### 2.7. Evaluation Metrics

We evaluated the beampattern optimization by comparing the optimized beampatterns produced by the different methods to the desired beampattern mask. Below, we describe the evaluation metrics used:

- Passband ripple;
- Beamwidth;
- Amplitude deviation.

#### 2.7.1. Passband Ripple

**Figure 4:**
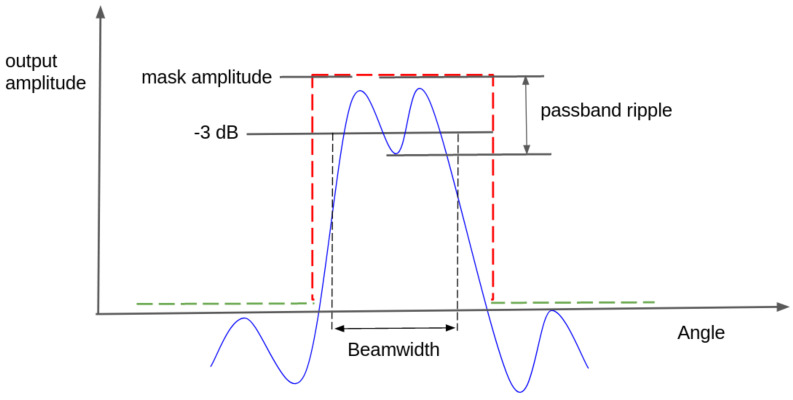
Example of beampattern mask (red and green lines) and an optimized beampattern response (blue line) with the evaluation measures of the passband ripple and beamwidth marked out. The red dotted line defines the mask of the main beam, whereas the green dotted line defines the max level for the allowed sidelobe level.

We employed the definition of a passband ripple from digital filter design, where the ripple is defined as fluctuations in the passband of the filter’s frequency magnitude response. In our case, the passband ripple was instead defined as the beampattern fluctuation within the angular interval for the main lobe of the desired beampattern mask ($BF_{tar}$). We describe the passband ripple as the maximum deviation from the desired beampattern mask in dB within the main lobe region; see Figure 4 for an illustrated example.

#### 2.7.2. Beamwidth

Here, we employed the definition of bandwidth from filter design. For a bandpass filter in the frequency domain, the bandwidth was defined as the interval between the two half-power points where the maximum amplitude response of the filter has decreased by 3 dB. Since we are working in the spatial domain with angular values on the x-axis instead of frequencies, we refer to the interval as beamwidth instead of bandwidth.

#### 2.7.3. Amplitude Deviation

Deviation from the optimized beampattern to the desired beampatterns was measured using the mean absolute error (MAE) within the angular interval of the main lobe of the desired beampattern:

(11) $$MAE=\sum_{i=1}^{n}\frac{|y_{i}-x_{i}|}{n}$$

$y_{i}$ are the values of the optimized beampattern, $x_{i}$ are the values of the desired beampattern, and *n* are the total number of data points.

### 2.8. Implementation Details

This project was implemented in python on a laptop computer with a Intel Xeon W-10855M processor. The python DCCP package [33], and CVXPY [19] were used.

## 3. Results

### 3.1. Experimental Setup

Our experiments considered transmission beampattern optimization for a linear array of 11 elements uniformly distributed with λ/2 spacing in between. We considered the elements to be monopole and omnidirectional. We then created two types of masks, a uniform and rectangular mask and a ramp-shaped mask, to represent the different use cases of sonar transmission beampatterns. We then compared our proposed optimization method to the method of convex optimization and DCCP as implemented in CVXOPT.

The same optimization scheme was used for the rectangular and the ramp-shaped beampatterns. For convex optimization, we used the cp.CVXOPT solver with max_iters= 10,000,000 and $reltol=1\times 10^{-19}$. For both DCCP and our iterative-convex method, we used the cp.SCS solver with max_iters=100,000 and max_iter=1024; otherwise, we used the preset default parameters. We measured the difference between the desired beampattern and the optimized beampatterns in terms of MAE, passband ripple, and beamwidth.

The following sections present the results from the beampattern optimization and the quantization analysis.

### 3.2. Rectangular Mask

**Figure 5:**
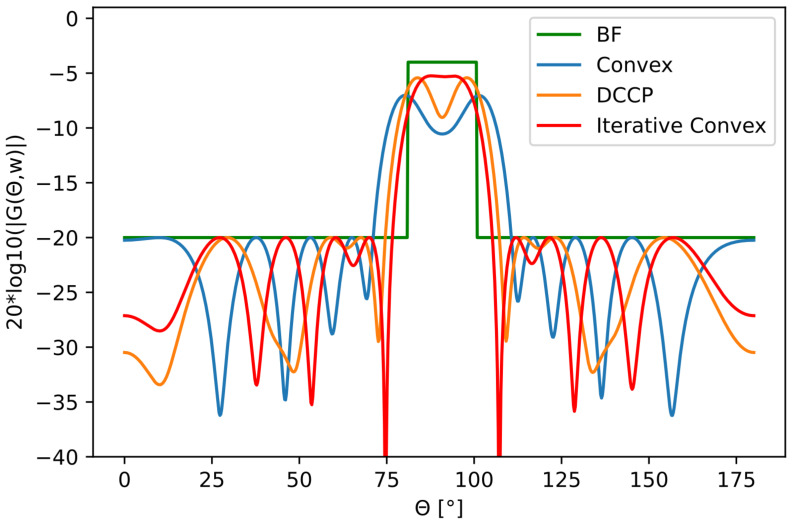
Uniform rectangular wide beampattern transmission mask in a $20^{\circ }$ interval together with optimized beampatterns.

**Figure 6:**
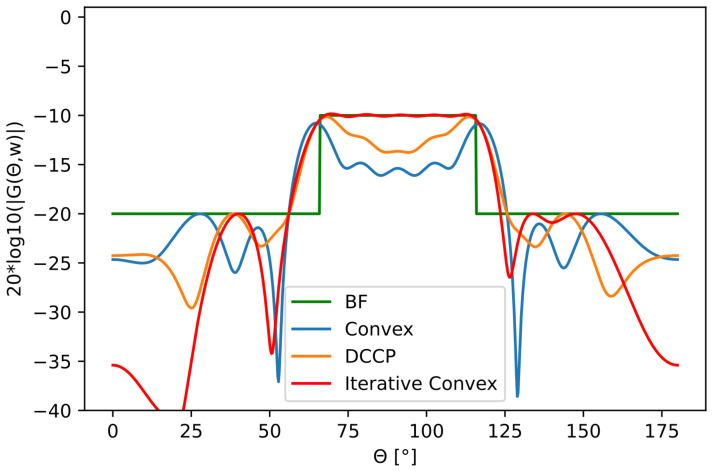
Uniform rectangular wide beampattern transmission mask in a $50^{\circ }$ interval together with optimized beampatterns.

We optimized two broad main lobes, one with a $20^{\circ }$ angular interval and the other one with a $50^{\circ }$. Figure 5 and Figure 6 show the results. The results for the optimized beampatterns were measured in MAE, passband ripple, and beamwidth defined by the −3 dB limit. The results are shown in Table 1 and Table 2, respectively.

### 3.3. Ramp-Shaped Mask

**Figure 7:**
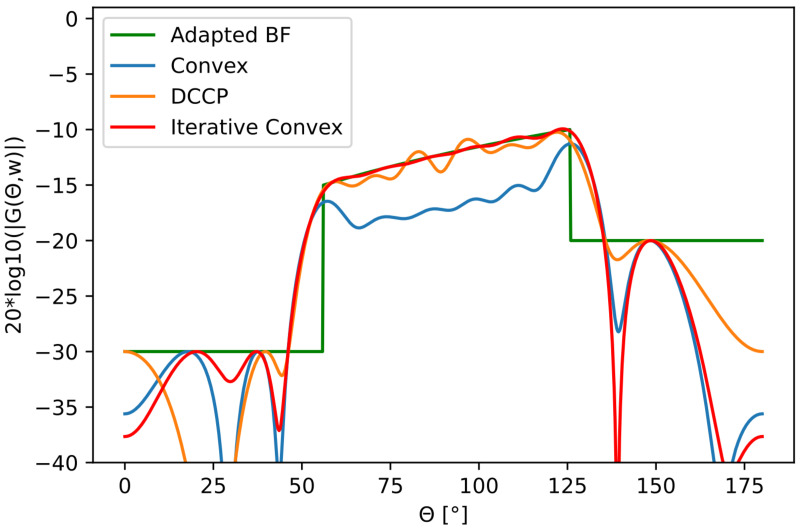
Ramp beampattern transmission mask in a $70^{\circ }$ interval together with optimized beampatterns.

The ramp-beampattern was designed for an SSS that insonifies a large swath of the seafloor (≈50 m). The SSS was assumed to be mounted on a vehicle having an altitude of around 15–20 m over the seabed. The beamwidth interval for the ramp-beampattern was $70^{\circ }$. The visual results for the desired beampattern and the optimized beampatterns are shown in Figure 7. The measured differences between the desired beampattern and optimized beampatterns are presented in Table 3.

### 3.4. Quantization Analysis

**Figure 8:**
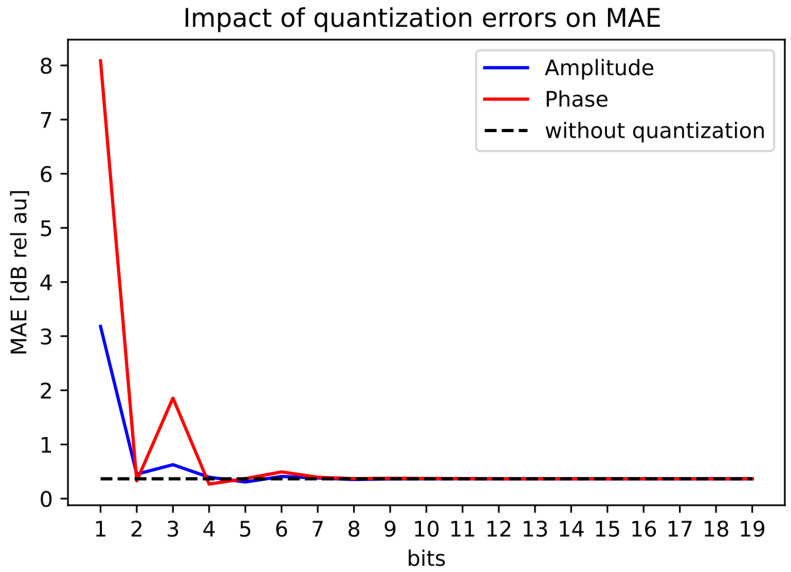
Quantization effect on the MAE for the ramped beampattern mask with BW = $70^{\circ }$.

**Figure 9:**
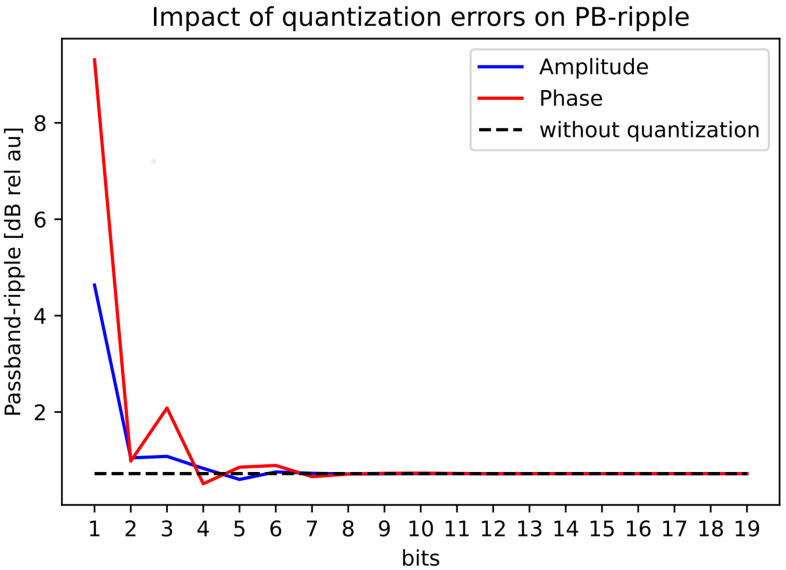
Quantization effect on the PB-ripple for the ramped beampattern mask with BW = $70^{\circ }$.

**Figure 10:**
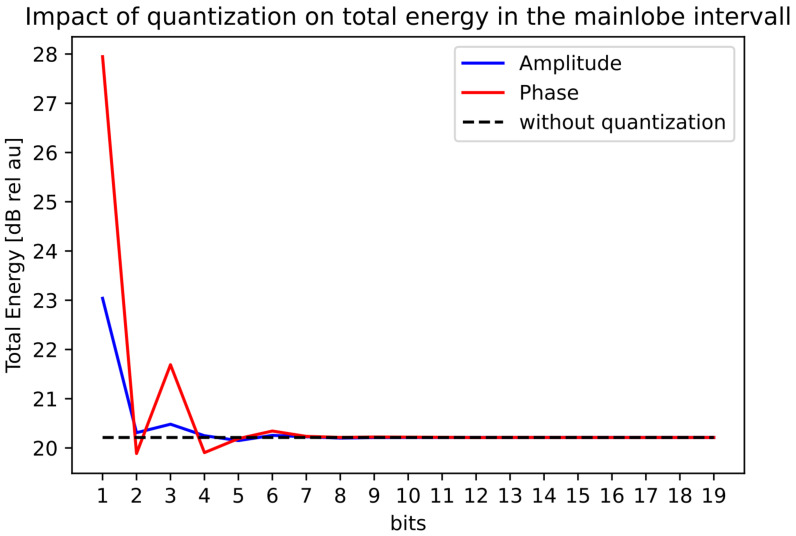
Quantization effect on the total energy in the main lobe interval for the ramped beampattern mask with BW = $70^{\circ }$.

**Figure 11:**
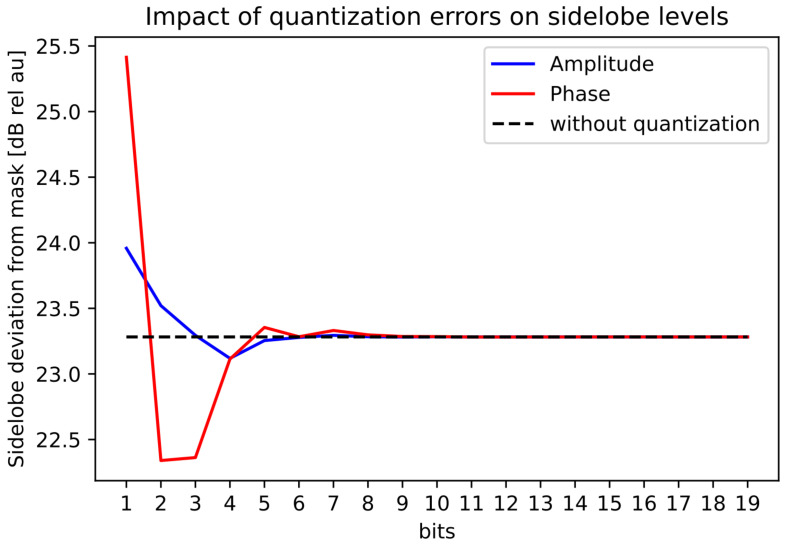
Quantization effect of the maximum sidelobe distance from the mask for the ramped beampattern mask with BW = $70^{\circ }$.

Using the ramp beam pattern from Figure 7, we used the iterative-convex optimization method and visualized the results of the effect of the limited accuracy of the bits in amplitude and phase for MAE, PB-ripple, the total energy in the mainbeam, and the sidelobe levels, see Figure 8, Figure 9, Figure 10 and Figure 11. The dotted black lines in the figures show the values achieved without quantization. Figure 8 shows the MAE for different quantization values in the main-lobe interval. Figure 9 shows the PB-ripple within the main lobe interval for different levels of quantization. Figure 10 shows the total measured energy in the main lobe interval for different levels of quantization. We can see that, at around 8-bits of quantization of the phase, the results converged to the same value as the ideal non-quantized case. The 8-bit phase quantization corresponded to an angular resolution of $1.4^{\circ }$. The quantization of the phase was more sensitive than the quantization of the amplitude. For the amplitude quantization, it was sufficient with only 5 bits to reach the non-quantization level of performance.

Finally, in Figure 11, we visualized the quantization effect on the sidelobes. Figure 11 follows the same trends for the quantization as the earlier figures.

## 4. Discussion

We presented an iterative-convex optimization method for wide beam transmission beamforming of narrowband signals. As convex optimization has not been used in the literature before for wide beam transmission beamforming of sonar beams, exploring these types of methods for this application is one of the main contributions of the paper. We compared the iterative-convex method to the standard convex optimization and DCCP.

Compared to the convex optimization the iterative-convex method and DCCP achieved optimized beampatterns closer to the desired beampattern masks. One drawback compared to convex optimization was the increase in computation time, but given that we do not require the beampatterns to be optimized online during operation, this should not be an issue for a variety of applications. It can be noted that the resulting main lobe beamwidth for the rectangular beampattern masks was wider than the desired mask beampattern. Allowing the desired beampattern mask to have complex values provided a significant improvement in the optimization results for both the DCCP and the iterative-convex method. When running the iterative-convex and DCCP for the same number of iterations, the iterative-convex method achieved optimized beampatterns closer to the desired beampattern masks. Thus, we conclude that the iterative-convex method was preferred before the DCCP. The better optimization performance of our proposed method is the second main contribution of this work. It could be interesting for future work to compare our proposed new iterative-convex optimization method to DCCP for other optimization problems.

Lastly, the effect of the quantization on the amplitude and phase was analyzed. It can be concluded that the quantization of the phase was more sensitive than the quantization of the amplitude. A quantization of at least 8 bits for the phase and 5 bits for the amplitude gave performance comparable to having no quantization.

An interesting continuation of this work could comprise of:

- Adjusting the method to work on broadband signals.
- Investigating the influence of the transmission elements experimentally.

## Figures and Tables

**Table 1:** Measurements of the beamwidth (BW), the passband-ripple (PB-ripple), and the MAE between the desired rectangular beampattern and the optimized beampatterns measured in the main lobe interval with BW = $20^{\circ }$. The best measurement values achieved are marked out in bold.

Method/Metric	MAE [dB]	BW [θ]	PB-Ripple [dB]
Convex	5.035	31.794	6.554
DCCP	2.675	24.283	5.044
Iterative-convex	**1.9164**	**20.278**	**4.475**

**Table 2:** Measurements of the beamwidth (BW), the passband-ripple (PB-ripple), and the MAE between the desired rectangular beampattern and the optimized beampatterns measured in the main lobe interval with BW = $50^{\circ }$. The best measurement values achieved are marked out in bold.

Method/Metric	MAE [dB]	BW [θ]	PB-Ripple [dB]
Convex	4.683	63.838	6.113
DCCP	2.121	58.831	3.766
Iterative-convex	**0.075**	58.831	**0.388**

**Table 3:** The measurements between the desired ramp beampattern and the optimized beampatterns measured in the main lobe interval with BW = $70^{\circ }$. The best measurement values achieved are marked out in bold.

Method/Metric	MAE [dB]	BW [θ]	PB-Ripple [dB]
Convex	4.254	70.347	5.221
DCCP	0.589	70.347	1.564
Iterative-convex	**0.095**	70.347	**0.606**

## Data Availability

Not applicable.
